# Evaluation and Validation of Plasma Proteins Using Two Different Protein Detection Methods for Early Detection of Colorectal Cancer

**DOI:** 10.3390/cancers11101426

**Published:** 2019-09-25

**Authors:** Megha Bhardwaj, Anton Gies, Korbinian Weigl, Kaja Tikk, Axel Benner, Petra Schrotz-King, Christoph H. Borchers, Hermann Brenner

**Affiliations:** 1 Division of Preventive Oncology, German Cancer Research Center (DKFZ) and National Center for Tumor Diseases (NCT), 69120 Heidelberg, Germany; megha.bhardwaj@nct-heidelberg.de (M.B.); anton.gies@nct-heidelberg.de (A.G.); petra.schrotz-king@nct-heidelberg.de (P.S.-K.); 2 Medical Faculty Heidelberg, University of Heidelberg, 69120 Heidelberg, Germany; 3 Division of Clinical Epidemiology and Aging Research, German Cancer Research Center (DKFZ), 69120 Heidelberg, Germany; k.weigl@dkfz-heidelberg.de (K.W.);; 4 German Cancer Consortium (DKTK), German Cancer Research Center (DKFZ), 69120 Heidelberg, Germany; 5 Division of Biostatistics, German Cancer Research Center (DKFZ), 69120 Heidelberg, Germany; benner@dkfz-heidelberg.de; 6 University of Victoria-Genome British Columbia Proteomics Centre, University of Victoria (UVic), Victoria, BC V8Z 7X8, Canada; christoph.borchers@mcgill.ca; 7 Department of Biochemistry and Microbiology, University of Victoria, Victoria, BC V8P 5C2, Canada; 8 Segal Cancer Proteomics Centre, Lady Davis Institute, Jewish General Hospital, McGill University, Montreal, QC H3T 1E2, Canada; 9 Gerald Bronfman Department of Oncology, Jewish General Hospital, McGill University, Montreal, QC H3T 1E2, Canada

**Keywords:** colorectal cancer, early detection, screening, biomarkers, prevention, proximity extension assay, LC/MRM-MS, proteomics

## Abstract

Objective: Plasma protein biomarkers could be an efficient alternative for population-based screening for early detection of colorectal cancer (CRC). The objective of this study was to evaluate and validate plasma proteins individually and as a signature for early detection of CRC. Methods: In a three-stage design, proteins were measured firstly by liquid chromatography/multiple reaction monitoring-mass spectrometry (LC/MRM-MS) and later by proximity extension assay (PEA) in a discovery set consisting of 96 newly diagnosed CRC cases and 94 controls free of neoplasms at screening colonoscopy. Two algorithms (one for each measurement method) were derived by Lasso regression and .632+ bootstrap based on 11 proteins that were included in both the LC/MRM-MS and PEA measurements. Additionally, another algorithm was constructed from the same eleven biomarkers plus amphireglin, the most promising protein marker in the PEA measurements that had not been available from the LC/MRM-MS measurements. Lastly the three prediction signatures were validated with PEA in independent samples of participants of screening colonoscopy (CRC (*n* = 56), advanced adenoma (*n* = 101), and participants free of neoplasm (*n* = 102)). Results: The same four proteins were included in all three prediction signatures; mannan binding lectin serine protease 1, osteopontin, serum paraoxonase lactonase 3 and transferrin receptor protein 1, and the third prediction signature additionally included amphiregulin. In the independent validation set from a true screening setting, the five-marker blood-based signature including AREG presented areas under the curves of 0.82 (95% CI, 0.74–0.89), 0.86 (95% CI, 0.77–0.92) and 0.76 (95% CI, 0.64–0.86) for all, early and late stages CRC, respectively. Conclusion: Two different measurement methods consistently identified four protein markers and an algorithm additionally including amphiregulin, a marker measured by PEA only, showed promising performance for detecting early stage CRC in an independent validation in a true screening setting. These proteins may be potential candidates for blood-based tests for early detection of CRC.

## 1. Introduction

Colorectal cancer (CRC) contributes immensely to the global burden of cancers with 1.85 million incident cases and approximately 880,000 deaths per year. CRC is the third most common cancer and second leading cause of cancer mortality globally [1]. There is increasing evidence that CRC incidence and mortality could be effectively reduced by screening [2,3,4,5]. Nevertheless, the participation rates in screening programs are often low due to inconvenience and invasiveness in endoscopy-based programs [6,7] and by reservation against collection, handling and storage of stool in stool test based screening programs [8]. The participation in population-based screening programs could potentially improve with minimally invasive blood-based tests [9].

The human proteome is estimated to consist of more than 20,000 proteins [10] and is being intensively explored for blood-based biomarker research. Multiplex platforms like liquid chromatography/multiple reaction monitoring-mass spectrometry (LC/MRM-MS) [11,12] and proximity extension assay (PEA) [13,14] can facilitate accurate simultaneous detection of numerous proteins in one go and have been used for blood-based biomarker research. Both LC/MRM-MS and PEA possess the ability of detecting even low-abundance markers with analytical sensitivity in the nanogram/mL to picogram/mL range.

Several blood-based protein marker signatures have been identified in previous years but only very few studies validated the signatures in an independent validation set and, so far, no study has repeated protein measurements in the same set of samples with different protein detection methods [15,16]. Hence, the current study was performed with an objective to evaluate and validate a protein biomarker signature for CRC early detection in a three-stage design. The proteins were firstly assayed in blood samples of CRC cases and controls using LC/MRM-MS in order to identify a promising multimarker algorithm. The same set of proteins was then measured separately using another highly sensitive method (PEA) on the same samples and an algorithm specific for the PEA measurements was derived. Finally, the estimates from both LC/MRM-MS and PEA were independently validated in prospectively collected samples of CRC and advanced adenoma (AA) cases and controls free of colorectal neoplasms that were exclusively recruited in a true screening setting.

## 2. Results

### 2.1. Characteristics of Study Population

The main characteristics of the populations from the discovery and the validation sets are shown in Table 1. The distribution of characteristics was largely similar across both the sets, with males representing around 60% of the study populations and the mean age was around 64 years in the discovery set and around 66 years in the validation set. The validation set included a higher proportion of stage I CRCs and a lower proportion of stage IV CRCs than the discovery set. Table 1 additionally includes the age and sex distribution of AA cases and controls in the entire screening population from which the AA cases and controls matched to CRC cases were drawn.

### 2.2. Individual Markers

The diagnostic performances of protein biomarkers cadherin 5 (CDH5), galectin 3 (Gal 3), insulin-like growth factor binding protein 2 (IGFBP2), mannan binding lectin serine protease 1 (MASP1), matrix metalloproteinase 9 (MMP9), myeloperoxidase (MPO), osteopontin (OPN), serum paraoxonase lactonase 3 (PON3), myeloblastin (PRTN3), SPARC protein (SPARC), transferrin receptor protein 1 (TR), and amphiregulin (AREG) are listed in Table 2. After adjustment for multiple testing, the number of proteins with significant differences (at *p*-value ≤ 0.05) between all stage CRC and controls were (*n* = 5, 7, and 3) using LC/MRM-MS and PEA on samples from the discovery set and PEA on the validation set, respectively. Area under the receiver operating characteristic (ROC) curve (AUC) s ≥ 0.60 were observed for five, seven, and three protein biomarkers using LC/MRM-MS and PEA on the discovery set and using PEA on the validation set, respectively. Of the eleven protein biomarkers quantified by both platforms, the best individual diagnostic performance in the discovery set was observed for PON3 with .632+ bootstrap adjusted AUC (AUC^BS^) of 0.72 (95% CI, 0.63–0.82) and 0.74 (95% CI, 0.66–0.84) for LC/MRM-MS and PEA measurements, respectively. In the validation set, the protein biomarker TR best detected CRC with an AUC^BS^ of 0.72 (95% CI, 0.64–0.85). As seen from Table 2 the diagnostic performance of all but three markers (MPO, OPN, and PRTN3) was similar for LC/MRM-MS and PEA measurements in the discovery set. The results of stage-specific diagnostic performances are summarized in Tables S1 and S2 for early and late stage CRC detection, respectively. As shown in these tables, in the validation set, the marker TR with AUCs^BS^ of 0.72 (95% CI, 0.58–0.90) and 0.71 (95% CI, 0.61–0.87) best detected early and late stage CRC cases, respectively.

### 2.3. Correlation Analysis

The correlation analysis for protein biomarkers measured across the same samples on the discovery set consisting of 190 participants (CRC = 96 and controls = 94) revealed that Pearson’s product-moment correlation coefficient was highest for PON3 (0.79) and was ≥0.6 for eight out of eleven biomarkers (Figure S1).

### 2.4. Multimarker Signatures

**Figure 1:**
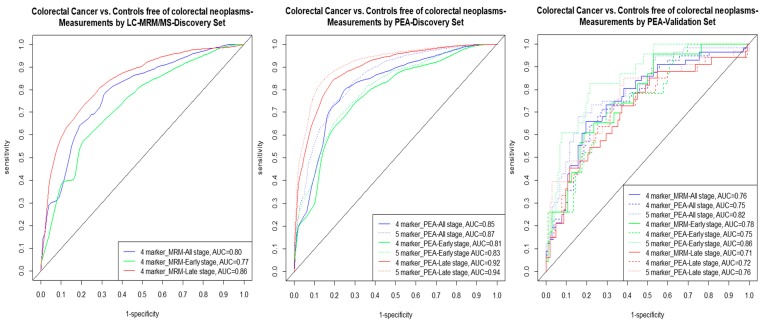
Comparison of the ROC curves for detecting (all/early/late) stage CRC vs. free of neoplasm controls with four and five-marker signatures. Abbreviations: AUC—area under the ROC curve; CRC—colorectal cancer; LC/MRM-MS—liquid chromatography/multiple reaction monitoring-mass spectrometry; PEA—proximity extension assay; ROC—receiver operating characteristics.

To assess whether the diagnostic performance of individual protein biomarkers can be improved further, we derived multimarker signatures for CRC detection. Figure 1 and Table 3 present an overview upon the diagnostic performance indicators.

When the least absolute shrinkage and selection operator (LASSO) with .632+ bootstrap was applied on LC/MRM-MS measurements of eleven protein biomarkers on the discovery set, a four-marker algorithm consisting of MASP1, OPN, PON3, and TR yielded AUCs^BS^ of 0.80, 0.75, and 0.84 for all, early, and late stage CRC detection, respectively. An identical four-marker signature was obtained when LASSO with .632+ bootstrap was applied on eleven biomarker measurements obtained using PEA on discovery set and AUCs^BS^ of 0.84, 0.79, and 0.90 were observed for all, early, and late stage CRC detection, respectively. When the estimates were validated independently on participants of screening colonoscopy for all stage CRC detection AUCs of 0.76 (95% CI, 0.67–0.85) and 0.75 (95% CI, 0.65–0.84) were observed for the LC/MRM-MS and PEA algorithms from the discovery set, respectively. The four-marker signature yielded AUCs of 0.78 (95% CI, 0.66–0.88) and 0.75 (95% CI, 0.63–0.86) for early stage CRC detection and AUCs of 0.71 (95% CI, 0.59–0.83) and 0.72 (95% CI, 0.59–0.83) for late stage CRC detection, for LC/MRM-MS and PEA algorithms, respectively.

When LASSO with .632+ bootstrap was applied on the eleven protein biomarkers and additionally AREG measurements of PEA in the discovery set, a five-marker signature (AREG, MASP1, OPN, PON3, and TR) was obtained and improved AUCs^BS^ of 0.87, 0.81, and 0.92 were observed for all, early, and late CRC detection. In the independent validation set comprising of participants of screening colonoscopy AUCs of 0.82 (95% CI, 0.74–0.89), 0.86 (95% CI, 0.77–0.92), and 0.76 (95% CI, 0.64–0.86) were observed for five-marker signature for all, early and late stage CRC detection, respectively. For the five-marker signature in the validation set, the sensitivities were 71%, 83%, and 58% at cutoffs yielding 80% specificity and 50%, 43%, and 45% at cutoffs yielding 90% specificity for all, early, and late stage CRC detection, respectively. The performances of all three algorithms for detection of participants with AA were similar and an AUC of 60 (95% CI, 0.51–0.69) was observed for the five-marker algorithm.

As described in Table 4, all five biomarkers had a wide variety of molecular functions and were involved in several biological processes. Additionally, Ingenuity Pathway Analysis (QIAGEN Inc., https://www.qiagenbioinformatics.com/products/ingenuity-pathway-analysis, version 01–10; Ingenuity Systems, Redwood City, CA, USA) was used to elucidate the interaction between these proteins (Figure S2), and it was observed that all these proteins interact at cellular or extracellular level in development of cancer despite their functional differences.

## 3. Discussion

In order to evaluate and validate promising plasma proteins for early detection of CRC and its precursors, we performed LC/MRM-MS and PEA on clinically recruited CRC cases and participants free of any colorectal neoplasm at screening colonoscopy. We validated the derived algorithms in an independent validation set, consisting of 56 CRC, 101 AA, and 102 individuals free of any colorectal neoplasm at screening colonoscopy, selected from over 9000 participants of the German screening colonoscopy program, recruited within the BLITZ (“Begleitende Evaluierung innovativer Testverfahren zur Darmkrebs-Früherkennung”) study. Evaluation of the performance of a five-marker signature including AREG, MASP1, OPN, PON3, and TR yielded AUCs of 0.82 (95% CI, 0.74–0.89), 0.86 (95% CI, 0.77–0.92), and 0.76 (95% CI, 0.64–0.86) for all, early, and late stage CRC detection, respectively, in the validation set.

The detection and quantitation of low-abundance biomarkers with low sample volume has been possible because of advancements in the field of proteomics. The peptides selected for the LC/MRM-MS resulted in convincing mass spectrometry signals and uniquely identified the target proteins. Moreover, using the triple-quadrupole mass spectrometer, high specificity is achieved by allowing only a selected peptide and then only a specific fragment to pass through the first and second quadrupoles, respectively. Relating to the PEA technology, the pair of oligonucleotide-labeled antibodies or probes have to be in close proximity and only this dual recognition of the target protein leads to initiation of an amplified signal detection. On account of these factors, both LC/MRM-MS and PEA are very target-specific methods. The technical assay sensitivity of the LC/MRM-MS is in the mid-high nanogram/mL range and for PEA, the analytical sensitivity is in the range of picogram/mL. In our study, protein measurements obtained by both technologies were highly correlated, and application of both technologies led to selection of the same four markers in the discovery set, which underlines the robustness and validity of our measurements. Nevertheless, even though the reproducibility of MRM and PEA platforms is good [17], replication of our findings using other quantification techniques that are easily applicable like ELISA should be aimed for in future studies. Apart from advanced technology used for detection and quantitation, the preanalytical processing of samples has been shown to influence the measurements in protein biomarker research [18,19,20]. In the current study, even though the participants were selected from three different studies, the collection, handling, processing, and storage of the samples across the three studies were performed with similar standardized operating procedure (SOPs). Although the samples from cases and controls free of neoplasms in the discovery set were collected over the three-year period between 2013 and 2016 and the participants selected in the validation set were recruited between 2005 and 2016, the storage time was comparable between cases and controls within both sets, and the replication of algorithms across studies with different storing times of samples further highlights the robustness and generalizability of the identified algorithms.

In recent years, several studies have identified blood-based protein marker panels and signatures, for which AUCs higher than 0.8 for were reported for detection of CRC. However, as identified in comprehensive systematic reviews, the participants in these studies were mostly recruited in clinical settings [15,16,21]. Two previous studies from our group had identified eight- and six-marker panels with AUCs for CRC of 0.76 [13] and 0.84 [22], respectively. However, unlike in the current study, the CRC cases in the validation sets of these studies had included clinically recruited cases. Another publication from our group where external validation included only participants recruited in a screening setting in the BLITZ study yielded an AUC of 0.82 for CRC detection [14] for a five-marker signature. In that study, the validation set had included a comparatively smaller number of screening colonoscopy participants with CRC (*n* = 41). In the current study, stage-specific algorithms were derived from a much smaller number of proteins that were included in each of two different highly target specific protein technologies, and the number of CRC cases in the validation set was higher than in our previous analyses. The limited diagnostic performance of the five-marker signature for detection of advanced adenomas (AUC = 0.60, 95% CI, 0.51–0.69), which is consistent with results from previous studies, underlines that major efforts are still needed to identify and validate biomarkers that would be able to detect these precursors with reasonable sensitivity.

Previous research on blood-based biomarkers other than proteins, such as COLOX (gene expression of 29 genes) [23], COLODETECT (four proteins + three phages) [24], CELTiC (four mRNAs) [25,26], and CANCERSEEK (16 genes + eight proteins) [27], yet again mostly included CRC cases that were recruited partially or completely in clinical settings and reported sensitivities of 79.5%, 89%, 79%, and 64.9% at specificities of 90%, 90%, 97%, and 99.1%, respectively. The diagnostic performance of these tests for early detection of CRC in screening setting samples is not known. Comparing such clinically recruited or mixed cases to healthy donors might result in spectrum bias [28,29], and these tests may benefit from validation in larger cohorts recruited exclusively in screening settings. In the PRESEPT clinical trial [30,31], DNA Epi proColon^®^ 2.0, the first FDA approved blood-based CRC screening test which is based on a Sept9gene methylation signature, was evaluated in a true screening setting and showed a sensitivity of 59% for stages I and II combined at 79% specificity in a true screening setting. With a sensitivity of 83% at a cutoff yielding 80% specificity in the validation set from participants of a true screening setting, the diagnostic performance of the five-marker signature derived in our current study is reasonably better for early detection of CRC than the first FDA-approved blood-based test for CRC screening.

The five proteins included in our signature are involved in different biological processes and have diverse molecular functions. Cytokines AREG and OPN have been identified previously as biomarkers with diagnostic potential for CRC [13,14,32,33,34]. The TR protein biomarker, which is involved in biological processes of host–virus interaction, and hydrolases PON3 and MASP1, have been recently associated with CRC detection. To our knowledge, this is the first time that the potential of these five biomarkers as a signature for early detection of CRC has been studied with an end validation in an independent set consisting of samples from participants of colonoscopy. Even though the diagnostic performance of the five markers is favorable compared to other blood-based tests, future research should address the possibility of further improvement of diagnostic performance of blood-based tests by combinations of these five markers with other proteomic and nonproteomic blood markers, with further validation in additional independent samples.

To our knowledge, this is the first study that identified, evaluated, and validated biomarkers for CRC early detection across two different platforms using a three-stage design. Strengths of our study include that we rigorously followed recommendations from previous research pertaining to blood-based protein biomarkers to adjust for over-optimism and to validate results obtained from samples of clinical cohorts in independent screening cohorts identifying preclinical cases from general population [15,16]. Consequently, in the current study we not only performed correction of over-optimism with the .632+ bootstrap method in the discovery set, but also externally validated our findings in an independent validation set that consisted of participants with CRC, AA, and without colorectal neoplasms at screening colonoscopy. Furthermore, we have measured the eleven protein biomarkers on two different independent detection methods (LC/MRM-MS and PEA), which are both highly sensitive, target-specific technologies and possess the ability of detecting even low-abundance markers using very low volumes of plasma. The diagnostic performance of the identified five-marker signature was fairly good for a blood-based test, with an AUC of 0.86 (95% CI, 0.77–0.92) for early stage CRC detection. Therefore, the identified plasma protein biomarkers are potential candidates for further research on blood-based tests for CRC screening and early detection. A major limitation is the still relatively small number of CRC cases in the validation set despite the very large screening population these cases were drawn from, which reflects the low prevalence of CRC in the screening population. The samples used in the current study are from a single point of time; therefore, future studies with assessment from two or more time points would help to evaluate intraindividual variability and to study the changes in proteome from benign to advancement into tumors. Additionally, given that participants suffering from cancers other than CRC were not included in the current study, future research including patients with other cancers or other systemic diseases would be important to determine to what extent the identified markers are CRC-specific.

## 4. Methods

### 4.1. Study Design

The protein biomarkers were assessed in a three-step approach. First measurements were performed in discovery set using LC/MRM-MS. Secondly, the performance was evaluated in the same samples using PEA and, lastly, the algorithms were validated in a validation set by using PEA again.

### 4.2. Study Population: Discovery Set

The discovery set included 96 CRC cases recruited prior to any therapeutic intervention from the iDa (“Durch innovative Testverfahren Darmkrebs früher erkennen”) study in hospitals in southwestern Germany between 2013 and 2016. We included 94 participants of screening colonoscopy as controls who were recruited in the ASTER (“Mit ASS Darmtumore früher erkennen”) study and were free of colorectal neoplasms. ASTER is a multicenter prospective randomized controlled trial (EudraCT No.2011-005603-32). Blood samples of ASTER participants were taken at recruitment in gastrointestinal practices in Germany from 2013 to 2016 [35]. The use of samples for early detection of CRC has been approved by the ethics committees of the Medical Faculty Heidelberg and from the responsible state medical boards, for both iDa and ASTER studies. The selection of study participants in the iDa and ASTER studies are reported in the complete Standards for the Reporting of Diagnostic accuracy (STARD) diagrams in Figures S3 and S4.

### 4.3. Study Population: Validation Set

**Figure 2:**
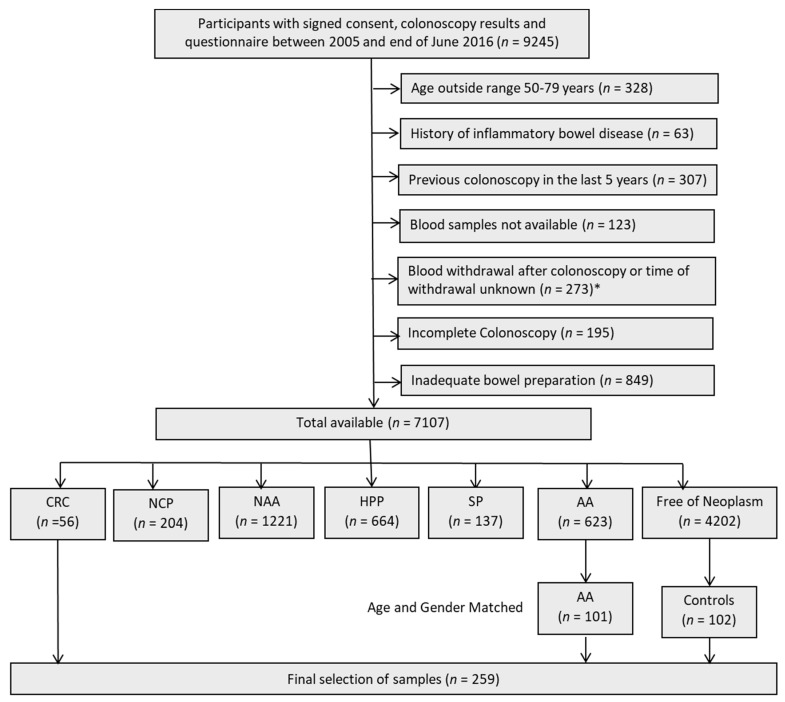
STARD (Standards for Reporting of Diagnostic Accuracy) flow diagram of the BLITZ Study. Abbreviations: AA—advanced adenoma; CRC—colorectal cancer; HPP—hyperplastic polyps; NAA—non advanced Adenoma; NCP—non classified polyp; SP—serrated poly. * The exclusion criterions for selection of CRC cases were not applicable after this point.

Blood samples for independent external validation of the algorithm were selected from participants of screening colonoscopy collected in the BLITZ (“Begleitende Evaluierung innovativer Testverfahren zur Darmkrebs-Früherkennung”) study. Details of the BLITZ study design have been reported previously [13,14,36,37,38]. Briefly, BLITZ is an ongoing prospective screening study of participants of the German screening colonoscopy program that is offered to the average risk population aged 55 years and older. Participants are recruited in 20 gastroenterology practices since the end of the year 2005. By the end of June 2016, out of 9425 participants in BLITZ, CRC, and AA had been detected in 56 and 623 participants, respectively. In the current study, validation of signatures identified in the discovery set using LC/MRM-MS and using PEA, were carried out in blood samples from 56 participants with CRC and 102 participants free of colorectal neoplasm at screening colonoscopy. Additionally, we enriched the study population with 101 participants with AA (advanced adenoma, defined as adenoma with >1 cm in diameter, tubulovillous or villous components, or high-grade dysplasia [39]. The controls free of neoplasms and AA participants were frequency matched to the CRC cases by sex and age. The use of samples from the BLITZ study for evaluation of early detection markers for CRC has been approved by the ethics committees of the Medical Faculty Heidelberg (S-178/2005), and of the physicians’ boards of Baden-Wuerttemberg (M118-05-f), Rhineland-Palatinate (837.047.06(5145)), Hesse (MC 254/2007) and Saarland (217/13). The STARD diagram showing selection of study participants from the BLITZ study is presented in Figure 2.

### 4.4. Sample Collection and Storage

Ethylenediaminetetraacetic acid (EDTA) plasma samples were collected before screening colonoscopy in ASTER and BLITZ and at first diagnosis of CRC before any treatment for cancer in iDa. After blood draw, samples were transported to the biobank at German Cancer Research Centre (DKFZ) in a cold chain. The plasma samples were then centrifuged between 2000–2500× *g* for 10 min and stored at −80 °C until the protein measurements. All the laboratory analyses were performed blinded with respect to disease status or findings at colonoscopy.

### 4.5. Laboratory Assays

Plasma samples were analyzed firstly in the discovery set using LC/MRM-MS for the targeted quantitation by peptide-based analysis for 270 unique proteins. The LC/MRM-MS analysis was performed at the Genome British Columbia Proteomics Centre, University of Victoria, BC, Canada (UVic). The assays utilized in the LC/MRM-MS possess high reproducibility and had been previously validated for their use in experiments following the Clinical Proteome Tumor Analysis Consortium (CPTAC) guidelines for assay development (https://assays.cancer.gov/) and the details of LC/MRM-MS have been published elsewhere [11,40]. Subsequently, 274 unique protein biomarkers were analyzed in the plasma samples of the discovery set and validation set using PEA, an antibody-based method, enhanced via primer extension and detected and quantified by qPCR, offered by Olink, Sweden. Olink’s Proseek^®^ Multiplex panels allow simultaneous analysis of 92 biomarkers and four internal controls in 1 µL samples [41] and the 276 proteins were distributed across the “Oncology II”, “Immune response” and “Cardiovascular III” Olink multiplex panels.

Of the 270 and 274 proteins analyzed by LC/MRM-MS and PEA, respectively, eleven proteins that overlapped between both measurements were included into the current study, namely, CDH5, Gal 3, IGFBP2, MASP1, MMP9, MPO, OPN, PON3, PRTN3, SPARC, and TR. Additionally, as AREG. was found to be the most promising marker out of the 274 biomarkers measured using PEA in the discovery set, it was also included in subsequent analyses.

### 4.6. Statistical Analysis

The data from LC/MRM-MS was visualized and examined with Skyline Quantitative Analysis software (version 4.1.1.18179, MacCoss Lab, Department of Genome, University of Washington, Seattle, WA, USA University of Washington) and the standard curve was used to calculate the peptide concentration in fmol/µL of plasma in the samples. The protein concentrations obtained from PEA were presented in the form of normalized protein expression (NPX).

The protein values obtained from both LC/MRM-MS and PEA were first compared for each individual biomarker between CRC and controls using Wilcoxon rank-sum test and correction for multiple testing by the Benjamini and Hochberg method [42]. For each individual protein AUC (95% CI) and sensitivity at the cutoff yielding 90% specificity was calculated. Additionally, in order to assess concordance between the eleven protein measurements using LC/MRM-MS and PEA on the samples in the discovery set, Pearson’s product-moment correlation coefficients were calculated.

In order to measure the diagnostic performance of multimarker combinations for detection of CRC, LASSO (Least absolute shrinkage and selection operator) regression models with .632+ bootstrap [43] to adjust for overfitting, were applied firstly to the eleven common protein biomarker measurements from LC/MRM-MS and subsequently, to the eleven protein measurements from PEA in the discovery set. Apart from the aforementioned two algorithms, another prediction algorithm was obtained by applying LASSO regression with .632+ bootstrap on twelve biomarker measurements of PEA on the discovery set. All three prediction algorithms were externally evaluated in the validation set that exclusively included participants of screening colonoscopy. The diagnostic performance of the derived algorithms was evaluated by calculating sensitivities at the cutoffs yielding 80% and 90% specificities and AUC not adjusted for overfitting with 95% CI, as well as AUC^BS^, i.e., the .632+ bootstrap adjusted AUC. All statistical analyses were performed with the statistical software R language and environment (version 3.5.0, R core team) [44]. For all tests, two-sided *p*-values of 0.05 or less were considered to be statistically significant.

## 5. Conclusions

Utilizing two different, highly sensitive target specific protein detection methods, we have identified a promising five-protein signature with diagnostic potential for early detection of CRC. The protein biomarkers AREG, MASP1, OPN, PON3, and TR exhibited diagnostic performance comparable to the only FDA-approved blood-based CRC screening test. Even though the diagnostic potential of the signature is not competitive with existing stool-based tests, such as fecal immunochemical tests for detection of fecal hemoglobin in screening settings [45], the biomarkers identified could contribute to the development of a promising blood-based test for population-based screening and early detection of CRC and its premalignant lesions.

## Figures and Tables

**Table 1:** Characteristics of the study population.

Group	Discovery Set		Validation Set			Participants of Screening Colonoscopy	
	iDa (Clinical) CRC	ASTER (Mostly Screening) Controls	BLITZ Matched Set (Screening)			BLITZ (Screening)	
			CRC	AA	Controls	AA	Controls
Total	*n* (%)	*n* (%)	*n* (%)	*n* (%)	*n* (%)	*n* (%)	*n* (%)
	96	94	56	101	102	623	4202
Age in years							
50–59	22 (23)	25 (27)	10 (18)	22 (21)	21 (21)	237 (38)	1916 (46)
60–69	41 (43)	44 (46)	28 (50)	49 (49)	50 (49)	247 (40)	1614 (38)
70–79	33 (34)	25 (27)	18 (32)	30 (30)	31 (30)	139 (22)	672 (16)
Mean	64.8	64.1	66.0	65.5	65.4	63.3	61.9
Median	65.0	66.0	65.0	65.0	65.5	62.0	60.0
SD	7.0	7.4	5.8	6.6	6.9	5.9	6.5
Gender distribution							
Male	59 (61)	55 (59)	36 (64)	65 (64)	66 (65)	393 (63)	1808 (43)
Female	37 (39)	39 (41)	20 (36)	36 (36)	36 (35)	230 (37)	2394 (57)
Stage distribution							
Stage I	17 (18)	-	17 (30)	-	-	-	-
Stage II	31 (32)	-	6 (11)	-	-	-	-
Stage III	22 (23)	-	26 (46)	-	-	-	-
Stage IV	26 (27)	-	(13)	-	-	-	-
Early stage (I/II)	48 (50)	-	23 (41)	-	-	-	-
Late stage (III/IV)	48 (50)	-	33 (59)	-	-	-	-

Abbreviations: AA—advanced adenoma; CRC—colorectal cancer; *n*—number; SD—standard deviation.

**Table 2:** Diagnostic performance of individual protein biomarkers for detecting CRC.

Protein Biomarkers	Discovery Set (LC/MRM-MS Measurements)					Discovery Set (PEA Measurements)				Validation Set (PEA Measurements)					
	*p*-val	*p*-val^adj^	AUC (95% CI)	AUC^BS^ (95% CI)	Se% at 90% Sp	*p*-val	*p*-val^adj^	AUC (95% CI)	AUC^BS^ (95% CI)	Se% at 90% Sp	*p*-val	*p*-val^adj^	AUC (95% CI)	AUC^BS^ (95% CI)	Se% at 90% Sp
CDH5	0.35	0.38	0.54 (0.46–0.62)	0.48 (0.36–0.61)	8	0.20	0.25	0.55 (0.47–0.64)	0.50 (0.36–0.64)	10	<0.05	<0.05	0.62 (0.54–0.71)	0.59 (0.50–0.73)	12
Gal 3	0.51	0.51	0.53 (0.45–0.61)	0.47 (0.36–0.59)	7	0.84	0.84	0.51 (0.43–0.59)	0.46 (0.37–0.55)	7	0.13	0.16	0.57 (0.48–0.67)	0.51 (0.33–0.67)	11
IGFBP2	<0.05	<0.05	0.61 (0.53–0.69)	0.58 (0.50–0.71)	24	<0.05	<0.05	0.61 (0.53–0.69)	0.58 (0.50–0.72)	21	0.43	0.46	0.54 (0.44–0.63)	0.48 (0.34–0.62)	10
MASP1	<0.001	<0.001	0.68 (0.61–0.76)	0.67 (0.58–0.78)	27	<0.001	<0.001	0.65 (0.57–0.72)	0.63 (0.53–0.75)	20	0.08	0.11	0.58 (0.49–0.68)	0.53 (0.37–0.68)	13
MMP9	0.24	0.33	0.55 (0.47–0.63)	0.50 (0.37–0.62)	10	0.32	0.36	0.55 (0.38–0.54)	0.50 (0.38–0.62)	10	0.05	0.08	0.59 (0.50–0.69)	0.55 (0.46–0.71)	16
MPO	0.06	0.11	0.58 (0.50–0.66)	0.47 (0.36–0.59)	8	<0.005	<0.005	0.63 (0.55–0.71)	0.60 (0.52–0.73)	17	0.53	0.53	0.53 (0.44–0.62)	0.46 (0.35–0.57)	6
OPN	<0.001	<0.001	0.64 (0.57–0.72)	0.62 (0.54–0.75)	26	<0.001	<0.001	0.75 (0.68–0.82)	0.73 (0.65–0.84)	35	<0.05	<0.05	0.62 (0.53–0.71)	0.59 (0.49–0.73)	18
PON3	<0.001	<0.001	0.73 (0.66–0.80)	0.72 (0.63–0.82)	32	<0.001	<0.001	0.75 (0.68–0.82)	0.74 (0.66–0.84)	43	0.05	0.08	0.59 (0.51–0.68)	0.54 (0.37–0.69)	11
PRTN3	0.13	0.21	0.56 (0.48–0.64)	0.51 (0.36–0.65)	10	<0.001	<0.001	0.64 (0.56–0.72)	0.61 (0.54–0.74)	18	0.06	0.09	0.59 (0.50–0.68)	0.49 (0.31–0.65)	5
SPARC	0.30	0.37	0.54 (0.46–0.63)	0.49 (0.37–0.61)	10	0.33	0.36	0.54 (0.46–0.62)	0.49 (0.36–0.62)	9	0.41	0.46	0.54 (0.45–0.63)	0.48 (0.36–0.61)	5
TR	<0.001	<0.001	0.67 (0.60–0.75)	0.66 (0.57–0.77)	35	<0.001	<0.001	0.70 (0.63–0.77)	0.69 (0.60–0.79)	36	<0.001	<0.001	0.74 (0.66–0.82)	0.72 (0.64–0.85)	33
AREG	-	-	-	-	-	<0.001	<0.001	0.79 (0.73–0.86)	0.78 (0.70–0.87)	54	<0.001	<0.001	0.72 (0.64–0.80)	0.70 (0.61–0.83)	35

Abbreviations: AUC—area under the receiver operating curve; AUC^BS^—.632+ bootstrap estimates of AUC; CRC—colorectal cancer; 95% CI—95% confidence interval; LC/MRM-MS—liquid chromatography/multiple reaction monitoring-mass spectrometry; PEA—proximity extension assay; Se—sensitivity; Sp—specificity. All proteins abbreviations: AREG—amphiregulin; CDH5—cadherin 5; Gal 3—galectin 3; IGFBP2—insulin like growth factor binding protein 2; MASP1—mannan binding lectin serine protease 1; MMP9—matrix metalloproteinase 9; MPO—myeloperoxidase; OPN—osteopontin; PON3—serum paraoxonase lactonase 3; PRTN3—myeloblastin; SPARC—SPARC protein; TR—transferrin receptor protein 1.

**Table 3:** Diagnostic performance of multimarker signatures for detecting CRC (all stages/early/late) and AA.

Protein Biomarkers Discovered in the Signatures	Discovery Set (LC/MRM-MS Measurements)				Discovery Set (PEA Measurements)				Validation Set (PEA Measurements) as in Screening Population		
	AUC^BS^	AUC	Se % at 80% Sp	Se % at 90% Sp	AUC^BS^	AUC	Se % at 80% Sp	Se % at 90% Sp	Weighted AUC (95% CI)	Se % at 80% Sp	Se % at 90% Sp
All stages CRC											
MASP1 + OPN + PON3 + TR ^#^	0.80	0.80	65	35	-	-	-	-	0.76 (0.67–0.85)	46	36
	-	-	-	-	0.84	0.85	73	42	0.75 (0.65–0.84)	46	36
AREG + MASP1 + OPN+ PON3 + TR ^§^	-	-	-	-	0.87	0.87	74	57	0.82 (0.74–0.89)	71	50
Early stages CRC											
MASP1 + OPN + PON3 + TR ^#^	0.75	0.77	56	38	-	-	-	-	0.78 (0.66–0.88)	43	30
	-	-	-	-	0.79	0.81	64	30	0.75 (0.63–0.86)	52	35
AREG + MASP1 + OPN + PON3 + TR ^§^	-	-	-	-	0.81	0.83	69	42	0.86 (0.77–0.92)	83	43
Late stages CRC											
MASP1 + OPN+ PON3 + TR ^#^	0.84	0.86	72	59	-	-	-	-	0.71 (0.59–0.83)	48	21
	-	-	-	-	0.90	0.92	85	67	0.72 (0.59–0.83)	55	33
AREG + MASP1 + OPN+ PON3 + TR ^§^	-	-	-	-	0.92	0.94	88	78	0.76 (0.64–0.86)	58	45
AA											
MASP1 + OPN+ PON3 + TR ^#^	-	-	-	-	-	-	-	-	0.58 (0.48–0.68)	28	19
	-	-	-	-	-	-	-	-	0.59 (0.49–0.68)	32	21
AREG + MASP1 + OPN + PON3 + TR ^§^	-	-	-	-	-	-	-	-	0.60 (0.51–0.69)	36	23

Abbreviations: AA—advanced adenomas; AUC—area under the receiver operating curve; AUC^BS^—.632+ bootstrap estimates of AUC; CRC—colorectal cancer; 95% CI—95% confidence interval; LC/MRM-MS—liquid chromatography/multiple reaction monitoring-mass spectrometry; PEA—proximity extension assay; Se—sensitivity; Sp—specificity. All proteins abbreviations: AREG—amphiregulin; MASP1—mannan binding lectin serine protease 1; OPN—osteopontin; PON3—serum paraoxonase lactonase 3; TR—transferrin receptor protein 1. Notes: ^#^—four markers based on 11 markers; ^§^—five markers based on 12 markers.

**Table 4:** Functions of proteins from the identified five-marker signature.

Biomarkers	Name	Uniprot ID	Molecular Function	Biological Process
AREG	amphiregulin	P15514	cytokine, growth factor	Cell–cell signaling, cell proliferation
MASP1	mannan-binding lectin serine protease 1	P48740	hydrolase, protease, serine protease	Complement activation lectin pathway, Immunity, Innate immunity
OPN/SPP1	osteopontin	P10451	cytokine	Biomineralization, Cell adhesion
PON3	serum paraoxonase lactonase 3	Q15166	hydrolase	Calcium, Metal-binding
TR/TFRC	transferrin receptor protein 1	P02786	host cell receptor for virus entry, receptor	Endocytosis, Host–virus interaction

## Supplementary Materials

The following are available online at https://www.mdpi.com/2072-6694/11/10/1426/s1, Figure S1: Correlation plot of the eleven protein markers measured with LC-MRM/MS and PEA in discovery set, Figure S2: Interaction of five identified biomarkers from all predictor models at subcellular level, Figure S3: STARD (Standards for Reporting of Diagnostic Accuracy) flow diagram showing selection of study participants enrolled in the iDa Study, Figure S4: STARD (Standards for Reporting of Diagnostic Accuracy) flow diagram showing selection of study participants enrolled in the ASTER Study during 2013–2016, Table S1: Diagnostic performance of individual protein biomarkers for detecting early stage (Stages I and II) CRC, Table S2: Diagnostic performance of individual protein biomarkers for detecting late stage (Stages III and IV) CRC.
