# Supplementary material for: Evaluation and Validation of Plasma Proteins Using Two Different Protein Detection Methods for Early Detection of Colorectal Cancer
Source: Cancers (Basel). 2019 Sep 25;11(10):1426. doi: 10.3390/cancers11101426 (PMC6826652; doi:10.3390/cancers11101426)
Supplement: Supplementary file 1 [file cancers-11-01426-s001.pdf]

cancers

MDPI

Supplementary Materials: Evaluation and Validation of Plasma Proteins Using Two Different Protein Detection Methods for Early Detection of Colorectal Cancer

Megha Bhardwaj, Anton Gies, Korbinian Weigl, Kaja Tikk, Axel Benner, Petra Schrotz-King, Christoph H. Borchers and Hermann Brenner

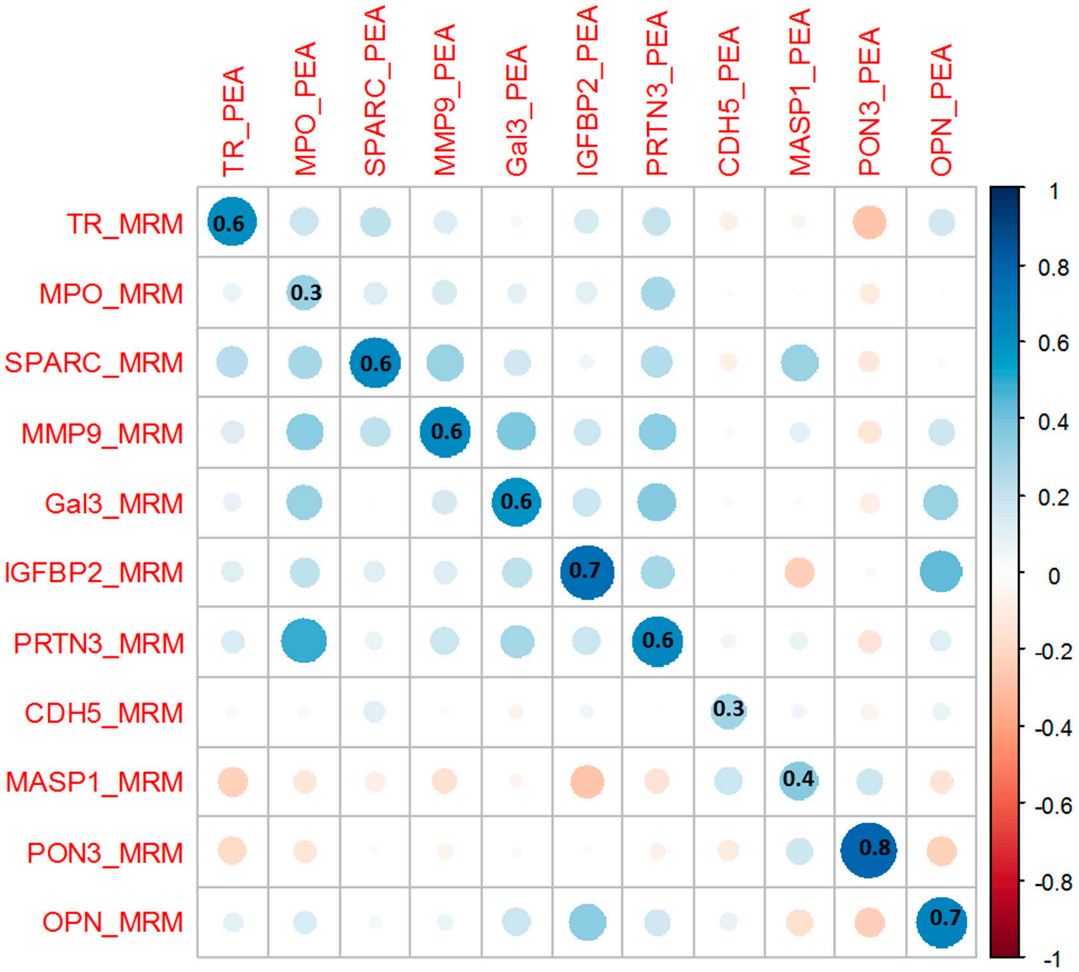

Figure S1. Correlation plot of the eleven protein markers measured with LC-MRM/MS and PEA in the discovery set. Abbreviations: CDH5—cadherin 5; Gal 3—galectin 3; IGFBP2—insulin like growth factor binding protein 2; LC/MRM-MS—liquid chromatography/multiple reaction monitoring-mass spectrometry; MASP1—mannan binding lectin serine protease 1; MMP9—matrix metalloproteinase 9; MPO—myeloperoxidase; OPN—osteopontin; PEA—proximity extension assay; PON3—serum paraoxonase lactonase 3; PRTN3—myeloblastin; SPARC—SPARC protein; TR—transferrin receptor protein 1.

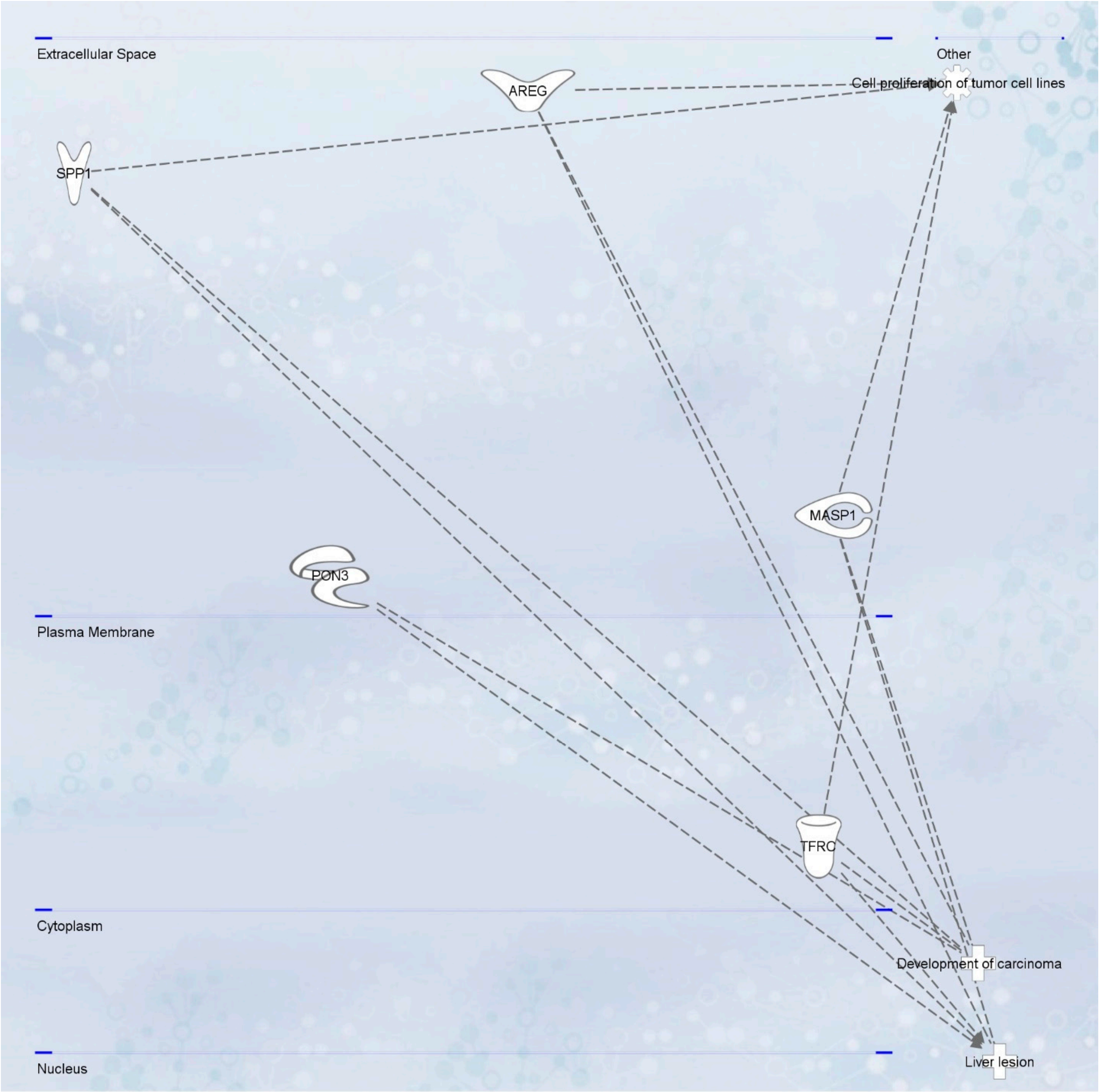

© 2000-2019 QIAGEN. All rights reserved.

Figure S2. Interaction of five identified biomarkers from all predictor models at the subcellular level. Abbreviations: AREG—amphiregulin; MASP1—mannan binding lectin serine protease 1; SPP1(OPN)—osteopontin; PON3—serum paraoxonase lactonase 3; TFRC(TR)—transferrin receptor protein 1.

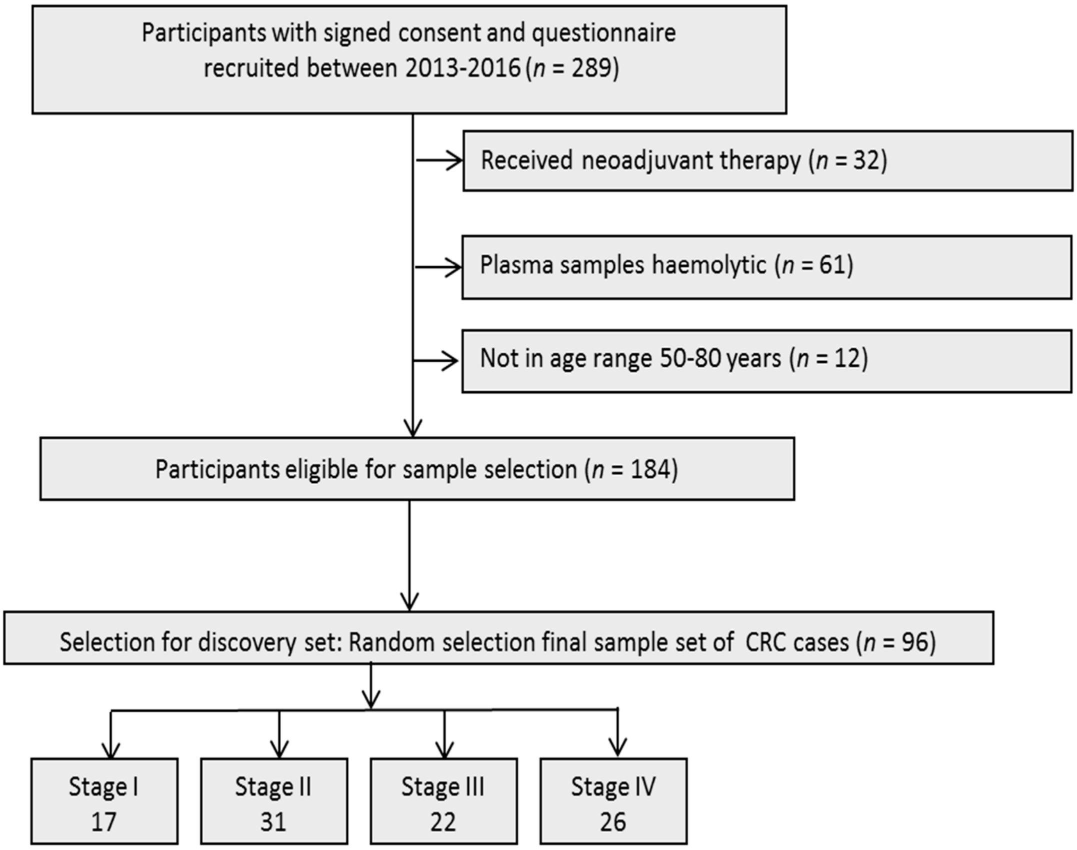

Figure S3. STARD (Standards for Reporting of Diagnostic Accuracy) flow diagram showing selection of study participants enrolled in the iDa Study. Abbreviation: CRC—colorectal cancer.

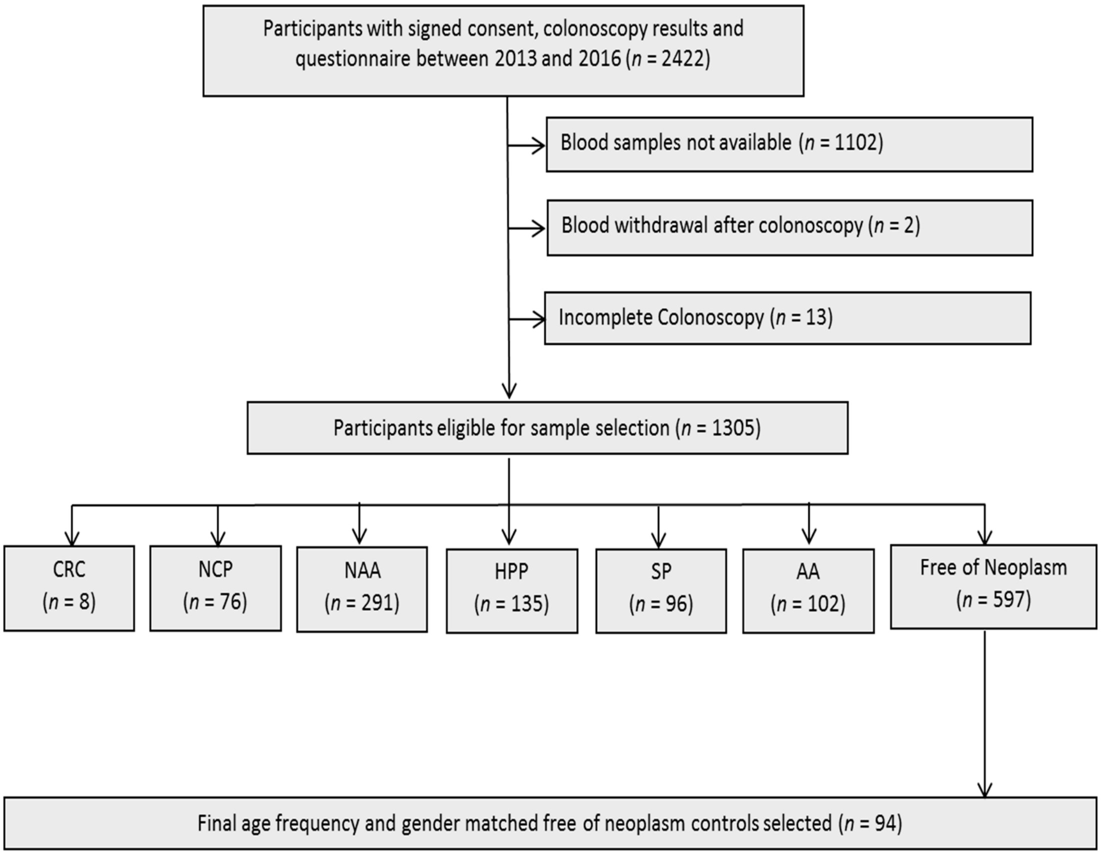

Figure S4. STARD (Standards for Reporting of Diagnostic Accuracy) flow diagram showing selection of study participants enrolled in the ASTER Study during 2013–2016. Abbreviations: AA—advanced adenoma; CRC—colorectal cancer; HPP—hyperplastic polyp; NAA—nonadvanced Adenoma; NCP—nonclassified polyp; SP—serrated polyp.

Table S1. Diagnostic performance of individual protein biomarkers for detecting early stage (Stage I and II) CRC.

Protein Biomarkers	Discovery Set (LC-MRM/MS Measurements)					Discovery Set (PEA Measurements)					Validation Set (PEA Measurements)				
	p-val	p-val^{adj}	AUC (95% CI)	AUC^{BS} (95% CI)	Se% at 90% Sp	p-val	p-val^{adj}	AUC (95% CI)	AUC^{BS} (95% CI)	Se% at 90% Sp	p-val	p-val^{adj}	AUC (95% CI)	AUC^{BS} (95% CI)	Se% at 90% Sp
CDH5	0.07	0.21	0.59 (0.50–0.69)	0.54 (0.43–0.71)	10	0.19	0.27	0.57 (0.47–0.67)	0.5 (0.32–0.66)	9	0.41	0.64	0.55 (0.43–0.68)	0.46 (0.26–0.66)	5
Gal 3	0.92	0.99	0.49 (0.4–0.59)	0.45 (0.35–0.58)	6	0.87	0.94	0.51 (0.41–0.61)	0.46 (0.35–0.59)	6	0.05	0.12	0.63 (0.51–0.75)	0.55 (0.3–0.77)	13
IGFBP2	0.67	0.93	0.48 (0.37–0.58)	0.47 (0.33–0.61)	12	0.86	0.94	0.49 (0.39–0.59)	0.46 (0.34–0.59)	7	0.60	0.65	0.54 (0.4–0.67)	0.45 (0.28–0.63)	7
MASP1	<0.05	0.05	0.63 (0.53–0.73)	0.59 (0.49–0.76)	17	<0.005	<0.01	0.65 (0.56–0.75)	0.62 (0.53–0.77)	16	0.50	0.65	0.54 (0.41–0.68)	0.45 (0.26–0.65)	6
MMP9	0.99	0.99	0.5 (0.40–0.60)	0.45 (0.34–0.57)	8	0.94	0.94	0.5 (0.39–0.60)	0.46 (0.34–0.57)	8	0.11	0.22	0.61 (0.48–0.74)	0.53 (0.34–0.77)	15
MPO	0.23	0.43	0.56 (0.46–0.67)	0.46 (0.33–0.61)	7	0.19	0.27	0.57 (0.47–0.67)	0.51 (0.32–0.67)	13	0.89	0.89	0.51 (0.40–0.62)	0.45 (0.33–0.61)	6
OPN	0.22	0.43	0.56 (0.46–0.65)	0.5 (0.34–0.65)	14	<0.005	0.005	0.66 (0.57–0.75)	0.63 (0.53–0.79)	17	<0.005	<0.01	0.71 (0.60–0.82)	0.68 (0.56–0.86)	24
PON3	<0.001	<0.001	0.73 (0.65–0.82)	0.72 (0.62–0.84)	33	<0.001	<0.001	0.73 (0.64–0.81)	0.71 (0.61–0.84)	35	0.26	0.45	0.58 (0.45–0.7)	0.49 (0.30–0.71)	9
PRTN3	0.84	0.99	0.51 (0.41–0.61)	0.47 (0.35–0.60)	6	0.08	0.14	0.59 (0.49–0.69)	0.52 (0.34–0.69)	12	0.60	0.65	0.53 (0.43–0.64)	0.43 (0.31–0.59)	5
SPARC	0.53	0.84	0.53 (0.43–0.64)	0.47 (0.33–0.62)	10	0.68	0.86	0.52 (0.41–0.63)	0.46 (0.32–0.61)	8	0.60	0.65	0.54 (0.41–0.66)	0.46 (0.30–0.64)	4
TR	<0.05	0.05	0.63 (0.53–0.73)	0.61 (0.49–0.77)	33	<0.05	<0.05	0.63 (0.53–0.73)	0.61 (0.48–0.76)	29	<0.001	<0.005	0.74 (0.63–0.86)	0.72 (0.58–0.90)	35
AREG	-	-	-	-	-	<0.001	<0.001	0.7 (0.61–0.80)	0.68 (0.58–0.82)	37	<0.005	<0.005	0.73 (0.60–0.85)	0.7 (0.56–0.89)	36

Abbreviations: AUC—area under the receiver operating curve; AUC^{BS}—.632+ bootstrap estimates of AUC; CRC—colorectal cancer; 95% CI—95% confidence interval; LC/MRM-MS—liquid chromatography/multiple reaction monitoring-mass spectrometry; PEA—proximity extension assay; Se—sensitivity; Sp—specificity. All proteins abbreviations: AREG—amphiregulin; CDH5—cadherin 5; Gal 3—galectin 3; IGFBP2—insulin like growth factor binding protein 2; MASP1—mannan binding lectin serine protease 1; MMP9—matrix metalloproteinase 9; MPO—myeloperoxidase; OPN—osteopontin; PON3—serum paraoxonase lactonase 3; PRTN3—myeloblastin; SPARC—SPARC protein; TR—transferrin receptor protein 1.

Table S2. Diagnostic performance of individual protein biomarkers for detecting late stage (Stage III and IV) CRC.

Protein Biomarkers	Discovery Set (LC-MRM/MS Measurements)					Discovery Set (PEA Measurements)					Validation Set (PEA Measurements)				
	p-val	p-val^{adj}	AUC (95% CI)	AUC^{BS} (95% CI)	Se% at 90% Sp	p-val	p-val^{adj}	AUC (95% CI)	AUC^{BS} (95% CI)	Se% at 90% Sp	p-val	p-val^{adj}	AUC (95% CI)	AUC^{BS} (95% CI)	Se% at 90% Sp
CDH5	0.81	0.81	0.51 (0.41–0.62)	0.45 (0.34–0.58)	6	0.42	0.46	0.54 (0.44–0.64)	0.48 (0.33–0.63)	7	0.003	0.01	0.67 (0.57–0.77)	0.63 (0.54–0.80)	13
Gal 3	0.24	0.29	0.56 (0.46–0.66)	0.50 (0.32–0.66)	13	0.62	0.62	0.53 (0.42–0.63)	0.46 (0.34–0.6)	8	0.54	0.54	0.53 (0.42–0.65)	0.47 (0.31–0.63)	9
IGFBP2	<0.001	<0.001	0.70 (0.60–0.79)	0.67 (0.56–0.82)	33	<0.001	<0.001	0.72 (0.62–0.81)	0.69 (0.58–0.83)	33	0.49	0.53	0.54 (0.42–0.66)	0.47 (0.30–0.64)	10
MASP1	<0.001	<0.001	0.74 (0.65–0.82)	0.72 (0.62–0.85)	33	0.01	0.01	0.64 (0.54–0.74)	0.61 (0.50–0.76)	22	0.06	0.10	0.61 (0.50–0.72)	0.55 (0.39–0.75)	15
MMP9	0.05	0.08	0.60 (0.50–0.70)	0.54 (0.39–0.71)	13	0.12	0.15	0.58 (0.48–0.68)	0.53 (0.42–0.70)	12	0.13	0.21	0.58 (0.47–0.7)	0.53 (0.40–0.72)	15
MPO	0.06	0.08	0.60 (0.50–0.69)	0.46 (0.33–0.59)	6	<0.001	<0.001	0.69 (0.60–0.78)	0.66 (0.56–0.81)	18	0.45	0.53	0.54 (0.43–0.65)	0.46 (0.32–0.61)	6
OPN	<0.001	<0.001	0.73 (0.64–0.82)	0.71 (0.61–0.85)	39	<0.001	<0.001	0.83(0.76–0.90)	0.82 (0.73–0.93)	54	0.36	0.50	0.55 (0.44–0.67)	0.49 (0.32–0.67)	12
PON3	<0.001	<0.001	0.72 (0.63–0.81)	0.71 (0.60–0.84)	28	<0.001	<0.001	0.77 (0.69–0.86)	0.76 (0.65–0.88)	49	0.06	0.10	0.61 (0.50–0.71)	0.54 (0.34–0.73)	9
PRTN3	<0.01	<0.05	0.62 (0.51–0.72)	0.54 (0.40–0.72)	12	<0.001	<0.001	0.69 (0.60–0.78)	0.66 (0.57–0.81)	20	<0.05	0.06	0.63 (0.53–0.72)	0.52 (0.28–0.72)	8
SPARC	0.29	0.32	0.55 (0.45–0.65)	0.49 (0.33–0.65)	10	0.25	0.29	0.56 (0.46–0.66)	0.49 (0.35–0.66)	6	0.46	0.53	0.54 (0.44–0.64)	0.46 (0.31–0.62)	6
TR	<0.001	<0.001	0.71 (0.62–0.81)	0.69 (0.58–0.85)	36	<0.001	<0.001	0.77 (0.69–0.85)	0.76 (0.66–0.87)	43	<0.001	<0.001	0.74 (0.64–0.84)	0.71 (0.61–0.87)	28
AREG	-	-	-	-	-	<0.001	<0.001	0.88 (0.82–0.94)	0.87 (0.79–0.96)	69	<0.001	<0.001	0.71 (0.61–0.81)	0.69 (0.57–0.85)	33

Abbreviations: AUC—area under the receiver operating curve; AUC^{BS}—.632+ bootstrap estimates of AUC; CRC—colorectal cancer; 95% CI—95% confidence interval; LC/MRM-MS—liquid chromatography/multiple reaction monitoring-mass spectrometry; PEA—proximity extension assay; Se—sensitivity; Sp—specificity. All proteins abbreviations: AREG—amphiregulin; CDH5—cadherin 5; Gal 3—galectin 3; IGFBP2—insulin like growth factor binding protein 2; MASP1—mannan binding lectin serine protease 1; MMP9—matrix metalloproteinase 9; MPO—myeloperoxidase; OPN—osteopontin; PON3—serum paraoxonase lactonase 3; PRTN3—myeloblastin; SPARC—SPARC protein; TR—transferrin receptor protein 1.

© 2019 by the authors. Licensee MDPI, Basel, Switzerland. This article is an open access article distributed under the terms and conditions of the Creative Commons Attribution (CC BY) license (http://creativecommons.org/licenses/by/4.0/).
